# Homologous mesenchymal stem cells promote the emergence and growth of pulmonary metastases of the rat osteosarcoma cell line UMR-106

**DOI:** 10.3892/ol.2014.2127

**Published:** 2014-05-08

**Authors:** PENG ZHANG, LING DONG, HUA LONG, TONG-TAO YANG, YONG ZHOU, QING-YU FAN, BAO-AN MA

**Affiliations:** 1 Department of Orthopedic Surgery, Tangdu Hospital, Fourth Military Medical University, Xi’an, Shaanxi 710032, P.R. China; 2 Department of Orthopedic Surgery, Urumqi General Hospital, Urumqi, Xinjiang 830000, P.R. China; 3 Department of Physiology, Fourth Military Medical University, Xi’an, Shaanxi 710032, P.R. China

**Keywords:** osteosarcoma, mesenchymal stem cells, vascular endothelial growth factor, metastasis, rat

## Abstract

Osteosarcoma (OS) is the most frequent primary bone sarcoma and tends to develop pulmonary metastasis. Studies have shown that mesenchymal stem cells (MSCs) are involved in OS growth and metastasis, but the mechanism remains unclear. The aim of the present study was to identify whether homologous MSCs could promote the growth and metastasis of OS in rats with a normal immune system. The OS cell line, UMR-106, which originally derives from a Sprague-Dawley (SD) rat-transplantable osteogenic sarcoma with an osteoblastic phenotype, has a strong carcinogenic capability and a high lung metastasis. Xenotransplanted models of UMR-106 with or without MSCs injected through the tibia (IT) or caudal vein (IV) were established. SD rats were randomly divided into six groups: Control, UMR-106 (IT), MSCs (IV), UMR-106 (IT) + MSCs (IV), UMR-106 (IV) and UMR-106 (IV) + MSCs (IV). Following injection, all rats were sacrificed at week 5, and the volume and quantity of metastatic sarcoma and the serum alkaline phosphatase levels were measured. There was no metastatic sarcoma in the liver, spleen and kidney in all groups. The rats in the MSCs (IV) + UMR-106 (IV) group showed a significantly higher volume and number of pulmonary metastatic tumors than those of the UMR-106 (IV) group. In pulmonary metastatic tissues, MSCs were found in the MSCs (IV) + UMR-106 (IV) group, but not in the UMR-106 (IT) + MSCs (IV) group. Notably, the expression of vascular endothelial growth factor (VEGF) was increased in the MSCs + UMR-106 cells co-culture system. The present study indicated that MSCs can significantly promote the pulmonary metastasis of the rat OS cell line, UMR-106, with a normal immune system, and VEGF was involved in MSC-promoted UMR-106 emergence and growth of pulmonary metastasis.

## Introduction

In the global population, metastasis is the most frequent and life-threatening complication associated with cancer (1). Metastasis leads to >90% of mortalities in cancer patients (1), and ~95% of patients who succumb to metastatic disease have metastasis in the lung, as indicated by autopsy (2). For osteosarcoma (OS), >80% of patients develop a recurrent disease within 2 years. When treated by surgery alone, more than half of patients will develop metastasis within 6 months (3). Studies have shown that MSCs promote the growth and pulmonary metastasis of breast cancer and OS (4,5). Several studies have also shown that the injection of MSCs into a vein promoted the metastasis established in subcutaneous or primary sites (6,7). In addition, a study showed that B16 melanoma cells transplanted into allogeneic mice did not form tumors unless co-injected with MSCs (8). Subcutaneous inoculation of COS1NR cells followed by intravenous injection of MSCs at weeks 3 and 5 significantly increased the number of lung nodules (9). It has also been demonstrated that MSCs enhance the survival of follicular lymphoma B cells derived from human tumors. Additionally, treating MSCs with tumor necrosis factor-α increased the protective effect of MSCs; however, the mechanism by which MSCs are involved in the regulation of tumor cells remains elusive (as reviewed in 8). MSCs appear to play a significant role in the adaptation of these traits by carcinoma cells, initiating carcinoma cell phenotypes (10). These studies also indicated that the tumor cells may interact with MSCs, and MSCs subsequently promote the established micrometastasis.

Having a strong carcinogenic capability, UMR-106 is an aggressive, poorly immunogenic OS cell line with an osteoblastic phenotype (11). In 2001, UMR-106 cells were first used in orthotopic implantation animal models by inoculation into the tibia of athymic mice (12). The present study aimed to investigate whether homologous MSCs could interact with UMR-106 cells and promote UMR-106 cell growth and pulmonary metastasis within a normal immune system. Various established animal models with or without co-injection of UMR-106 cells and homologous MSCs were also compared. The aim of the present study was to explore the function of MSCs in the pulmonary metastasis of UMR-106 cells and the possible underling mechanisms of MSCs in promoting the emergence of UMR-106 cells, as well as the growth of pulmonary metastasis in rats with a normal immune system.

## Materials and methods

### OS cell line

The UMR-106 cell line, syngenic to Sprague-Dawley (SD) rats, was purchased from the American Type Culture Collection (Manassas, VA, USA). The UMR-106 cells were cultured in Dulbecco’s modified Eagle’s medium (DMEM) in 10% fetal bovine serum (FBS) (Sigma Aldrich, St. Louis, MO, USA) supplemented with L-glutamine (2 mM; Sigma Aldrich), penicillin (100 U/ml; Sigma Aldrich) and streptomycin (100 μg/ml; Sigma Aldrich), and confirmed to be mycoplasma-free by routine testing.

### Cell culture of MSCs

Male SD rats, 2 weeks old, were purchased from the Laboratory Animal Research Center of the Fourth Military Medical University (Xi’an, China). The rats were maintained in micro-isolator cages under specific pathogen-free conditions. The temperature was maintained at 24°C and the animals were exposed to a 24-h circadian rhythm with free access to water and food. The study was previously approved by the Fourth Military Medical University Ethics Committee for Animal Research. The bone marrow was aseptically collected and subsequently cultured using whole-marrow differential adherence methods (12). MSCs were obtained by multiple digestions and passages. MSCs were identified by the cellular surface marker expression [i.e., cluster of differentiation 29 (CD29), CD34, CD45 and CD90] using flow cytometry (13). Third generation MSCs were used in subsequent experiments and were pre-labeled with 4 μg/ml chloromethyl-dialkylcarbocyanine (CM-Dil; Invitrogen Life Technologies, Carlsbad, CA, USA) for 5 min at 37°C in pre-warmed phosphate-buffered saline (PBS), followed by incubation for 15 min at 4°C before the implantation experiment.

### Implantation technique

Four-week-old male SD rats were divided into six groups at random, with or without injected UMR-106 into the tibia (IT) or the caudal vein (IV) and with or without injection of MSCs into the caudal vein (IV). All animals were subsequently anesthetized intraperitoneally with 10% chloral hydrate at a dosage of 0.1 ml/30 g body weight and the operative field was prepared with iodine and draped. Orthotropic implantation models of the rats were performed by IT injection directly into the rats with a syringe (25-gauge needle) for inoculation with UMR-106 cells. Respectively, UMR-106 cells (1×10^7^ cells in 100 μl) were injected intraosseously into the proximal part of the tibia shaft. The tumor size was determined by measuring the largest and smallest diameter. The metastatic tumor volume was calculated according to the following formula: Tumor volume (mm^3^) = [largest diameter (mm) × smallest diameter (mm)^2^]/2.

### Histopathological and immunohistochemical examination

Following fixation in buffered isotonic formaldehyde (100 ml of 37% formaldehyde solution, 900 ml distilled water, 4 g monobasic sodium phosphate and 6.5 g dibasic sodium phosphate), implantation tumor and lung sections were embedded in paraffin for 24 h. Samples were then immersed in 70% alcohol and stained with hematoxylin-eosin. The samples were examined by a pathologist in a blinded manner (12).

Co-cultured MSCs and UMR-106 cells in a suspension of 1×10^4^ cells (MSCs:UMR-106 cell ratio, 1:1) in 2 mL DMEM and 10% FBS were added into each dish. Following incubation for 12 h, the medium was replaced with DMEM and 1% FBS. Subsequently, after incubation for 24 h, the cells were washed with PBS, fixed with 4% paraformaldehyde for 30 min, and then prepared for histopathological and immunohistochemical examination. Each experiment was performed in triplicate. Cell climbing slices were treated with 3% hydrogen peroxide in methanol for 10 min to inactivate endogenous peroxidases and were then treated with a VEGF rabbit anti-mouse, anti-rat and anti-human polyclonal primary antibody (Abcam, Cambridge, MA, USA) overnight at 4°C. Subsequent to rinsing with PBS, the cell climbing slices were treated for 20 min with pre-diluted biotin-conjugated broad-spectrum immunoglobulin G polyclonal goat anti-rabbit, and anti-mouse secondary antibody (SBS Genetech Co., Ltd., Beijing, China), and then visualized using streptavidin-conjugated horseradish peroxidase provided with the Real Envision Detection kit (SBS Genetech Co., Ltd.) following instructions specified by the manufacturer.

### ELISA assay

To determine the secretion of VEGF in the supernatants from UMR-106, MSCs or co-cultured UMR-106 cells + MSCs, cells were plated in medium containing 1% FBS. After the cells were cultured for 48 h, the supernatants were collected according to the manufacturer’s instructions. The media were analyzed by a commercially available sandwich VEGF ELISA kit (Invitrogen Life Technologies). Assays were performed in quadruplicate. Results were normalized for the number of producing cells and reported as picograms of VEGF in 1×10^6^ cells per 48 h.

### Statistical analysis

SPSS 11.0 (SPSS, Inc., Chicago, IL, USA) was used for data variation analysis. Data were obtained from at least three independent experiments and presented as the mean ± standard deviation. Comparisons between two groups were performed with Student’s t-test, and the statistical significance of mean differences among multiple groups was obtained by analysis of variance followed by Dunnett’s post-hoc test. P<0.05 was considered to indicate a statistically significant difference.

## Results

### OS pulmonary metastasis is promoted by co-injection with MSCs

The development of OS metastasis was tested in response to MSCs to study the metastasis interaction between OS and MSCs and the underlying mechanism. Injection of UMR-106 cells with or without injection of MSCs through the caudal vein resulted in the tumors of the leg expanding vigorously with a time-lapse in the first 3 weeks. The tumor size was measured and calculated weekly post-inoculation, as in our previous study (12). There were no metastatic sarcomas in the liver, spleen and kidney for all groups. The pulmonary tumor metastatic rate is shown in Table I. Data were obtained from the measurement of the pulmonary metastasis rate of the UMR-106 cells. It was shown that there were no metastatic tumors in the control group, which were injected with normal saline solution, and that there were also no metastatic tumors in the MSC group. While in the other four groups, the number of rats having macroscopic and microscopic visible tumors increased. In the UMR-106 (IT) + MSCs (IV) group, 6 out of 10 rats had metastatic tumors. However, the number of rats with metastatic tumors (10/10) in the UMR-106 (IV) and UMR-106 (IV) + MSCs (IV) groups was identical.

**Figure 1:**
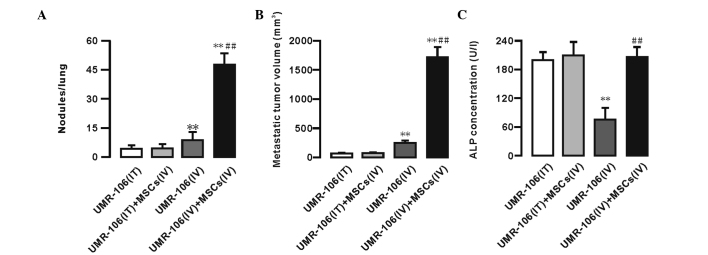
Pulmonary metastasis of UMR-106 cells *in vivo* in response to co-injection with MSCs. (A) Number of nodules per lung, (B) metastatic tumor volume and (C) ALP levels in the blood. ^*^P<0.05 and ^**^P<0.01, compared with the UMR-106 cells (IT) group. ^#^P<0.05 and ^##^P<0.01, compared with the UMR-106 cells (IV) group. Results are expressed as the mean ± standard deviation. ALP, alkaline phosphatase; IT, injected through tibia; IV, injected through caudal vein; MSCs, mesenchymal stem cells.

Furthermore, 5 weeks after the injection, the metastatic tumor nodules and volume per lung were measured. No significant differences in the number of metastatic tumor nodules and the metastatic tumor volume were identified between the UMR-106 (IT) and UMR-106 (IT) + MSCs (IV) groups. However, an increased number of metastatic tumor nodules and an enhanced metastatic tumor volume was observed in the UMR-106 (IV) + MSCs (IV) group (Fig. 1A and B). The data showed that the number of metastatic tumor nodules in the UMR-106 (IV) + MSCs (IV) group was significantly increased compared with that in the UMR-106 (IV) group (47.84±5.51 vs. 8.63±3.70; n=10; P<0.01). The metastatic tumor volume in the UMR-106 (IV) + MSCs (IV) group was significantly increased compared with that in the UMR-106 (IV) group (1737.4±199.61 vs. 251.84±56.04; n=10; P<0.01). The primary tibia tumor volume of the UMR-106 (IT) + MSCs (IV) group was greater than that of the UMR-106 (IT) group before the third week, but there was no difference between the UMR-106 (IT) and UMR-106 (IT) + MSCs (IV) groups in the fifth week (data not shown). Furthermore, the levels of alkaline phosphatase (ALP) in the blood serum were measured at week 5 to determine the progression of OS metastasis. The data showed that the serum ALP levels were not significantly different between the UMR-106 (IT) and UMR-106 (IT) + MSCs (IV) groups (198.39±16.92 vs. 208.04±30.71 U/l; n=10; P>0.05). In the UMR-106 (IV) group, the ALP levels were significantly decreased compared with those in the UMR-106 cells (IT) group, whereas the ALP levels were significantly enhanced in the UMR-106 (IV) + MSCs (IV) group compared with those in the UMR-106 (IV) group (205.29±23.59 vs. 75.12±24.12; n=10; P<0.01). However, there were no significant differences between the UMR-106 (IV) + MSCs (IV) group compared with the UMR-106 (IT) and UMR-106 (IT) + MSCs (IV) groups (Fig. 1C).

### MSCs increase in the OS tumor pulmonary metastatic site

**Figure 2:**
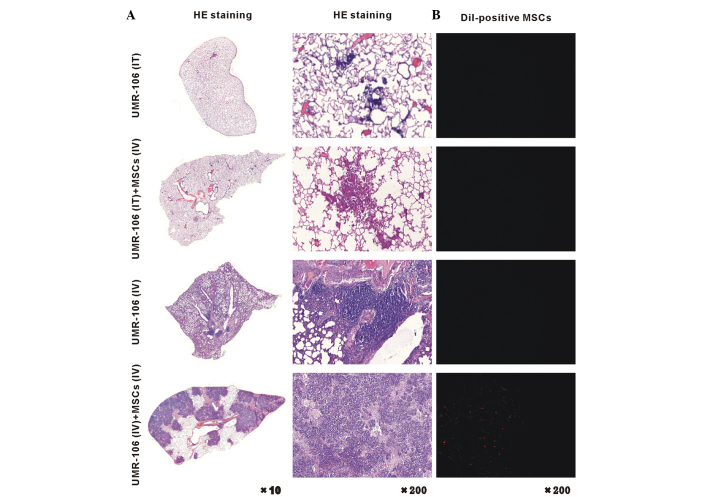
Pulmonary metastasis of UMR-106 cells in response to co-injection with Dil-positive MSCs. (A) Pathological analysis (HE staining; magnification, ×10 and ×200). (B) Fluorescence microscopy with red fluorescence and MSCs labeled with Dil (magnification, ×200). Dil, dialkylcarbocyanine; MSCs, mesenchymal stem cells; HE, hematoxylin and eosin; IT, injected through tibia; IV, injected through caudal vein.

Pathology results of the distribution of OS pulmonary metastatic site are shown in Fig. 2. The pulmonary metastatic OS was significantly increased in the UMR-106 (IV) + MSCs (IV) rats, compared with that in the UMR-106 (IT) + MSCs (IV) and UMR-106 (IV) rats (Fig. 2, left panel). Five weeks after injection of the CM-Dil-labeled MSCs, which can stain the MSC cell membrane red, an enhanced large portion of MSCs was found in the lung of the UMR-106 (IV) + MSCs (IV) group (Fig. 2, right panel). We propose that the UMR-106 cells were driven to undergo pulmonary metastasis by components that were secreted by MSCs, or that chemoattraction caused the UMR-106 cells and MSCs to intricately interact, resulting in the development of pulmonary metastasis. Overall, these observations indicate that the development and progression of OS pulmonary metastasis were promoted in response to MSCs.

### VEGF expression and secretion is enhanced in the MSCs and UMR-106 cells co-culture system

**Figure 3:**
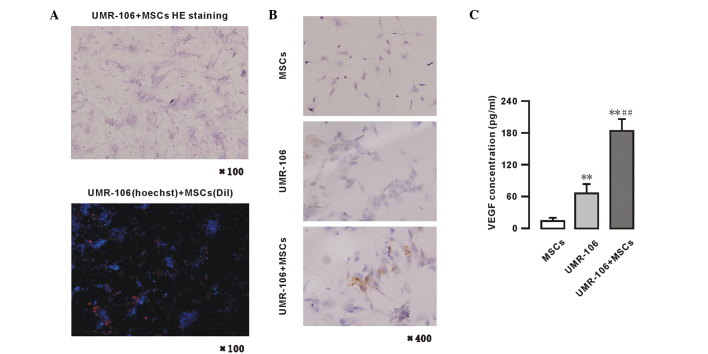
VEGF protein expressed in MSCs and UMR-106 cells in a co-culture system. (A) Pathological (HE staining; magnification, ×100) and fluorescence microscopy (blue, UMR-106 cells labeled with Hoechest; and red, MSCs labeled with Dil; magnification, ×100) analyses of a co-culture of UMR-106 cells and MSCs. (B) Immunohistochemistry for VEGF of UMR-106 cells and MSC colonies (magnification, ×400); (C) VEGF secretion in MSCs and UMR-106 cells, as determined by ELISA. Results are expressed as the mean ± standard deviation. ^*^P<0.05 and ^**^P<0.01, vs. the MSC group. ^#^P<0.05 and ^##^P<0.01, vs. the UMR-106 group. VEGF, vascular endothelial growth factor; MSCs, mesenchymal stem cells; HE, hematoxylin and eosin; Dil, dialkylcarbocyanine.

Growth of the UMR-106 cells and MSCs in co-culture system is shown in Fig. 3A. These UMR-106 cells showed a colony-like growth and the MSCs distributed between the colonies in a dense area. In the fluorescence microscopy images, the blue fluorescence were the UMR-106 cells labeled with Hoechest, and the red fluorescence were the MSCs labeled with Dil (x100). The expression of VEGF was also analyzed in the UMR-106 cells and MSCs by immunohistochemistry. Positive immunohistochemical staining for VEGF is shown in Fig. 3B. The VEGF protein was detected in the cytoplasm and membrane of MSCs and/or UMR-106 cells. The basal VEGF expression of MSCs and UMR-106 cells was low. However, in the UMR-106 + MSCs co-culture model system, when the two cells were co-cultured for 48 h, an increase in VEGF expression was observed. The levels of VEGF were also measured in the supernatants of the MSCs and UMR-106 cells co-culture system by ELISA (Fig. 3C) in the following groups: UMR-106 cells (1.0×10^6^ cells) alone, MSCs (1.0×10^6^ cells) alone and co-culture of UMR-106 cells + MSCs (0.5×10^6^ cells each). The data showed that an extremely low level of VEGF was secreted in cultured MSC supernatants, while in the UMR-106 cells group, the VEGF levels were significantly higher compared with the MSCs group (66.23±17.85 vs. 14.04±5.97 pg/1×10^6^ cells/48 h; n=4; P<0.01). However, in the supernatants of the MSCs co-cultured with UMR-106 cells group, the concentration of VEGF was significantly increased (184.45±22.44 pg/1×10^6^ cells/48 h; n=4; P<0.01) compared with the MSCs or UMR-106 cells group (Fig. 3C).

## Discussion

The present study showed that homologous MSCs promoted the pulmonary metastasis significantly subsequent to UMR-106 entering into circulation in the SD rat model, and MSCs were present in the pulmonary metastatic nodules. In addition, the UMR-106 cells and MSCs expressed little VEGF separately, but UMR-106 cells and MSCs expressed high levels of VEGF in a mixed culture. These results demonstrate that the interaction with MSCs causes the survival of UMR-106 cells and establishes metastasis in pulmonary parenchyma.

The cross-talk between tumor cells and the surrounding peri-tumoral stroma has been studied recently (14). The contribution of MSCs is believed to regulate carcinoma cell growth and motility (15). The homologous Dil-labeled MSCs were found in the metastatic colonies and MSCs increased the metastatic nodules in the lung (Fig. 2). However, other studies have also shown that prior to dissemination of the metastatic tumor cells the environment of the lung was altered in mice bearing subcutaneous metastatic melanomas or lung carcinomas (16–18). In these studies, by directing the recruitment of bone marrow-derived cells to the lungs, the tumors effected alterations in the distant lung parenchyma, in which disseminated tumor cells subsequently settled.

Metastasis is a cascade of molecular and cellular events, which involve tumor cell intravasation, transport and immune evasion in the circulatory system; arrest at a secondary site; extravasation; and finally colonization and growth (19). Once the cancer cells have entered the blood circulation, the number of cancer cells that eventually generate metastatic foci is even less (20,21) The possible mechanisms underlying the tumor and host MSCs interactions are associated with the steps of the metastasis. These include MSCs chemoattracted to UMR-106 cells that then become trapped UMR-106 cells in the circulation. The two types of cells interact with each other and express VEGF and achieve metastasis in pulmonary parenchyma. Notably, bone marrow-derived inflammatory cells have been found in elevated concentrations in the blood of patients with cancer (22). VEGF is one of these factors, which is secreted by tumor-associated inflammatory cells and fibroblasts, and acts pleiotropically to affect tumor cell proliferation, invasion and angiogenesis (5). The data of the present study showed that MSCs and UMR-106 cells expressed a low level of VEGF separately, but their mixed colonies expressed a high level of VEGF. This indicates that they interacted with each other in the mixed culture system and also upregulated the expression of VEGF. OS with lung metastasis has been reported to exhibit a high expression of VEGF (18,23,24,25). Our previous study showed that VEGF could determine the endothelial cell activation, proliferation and migration (26). VEGF is also a known OS angiogenesis inducer (24). OS with lung metastasis has been reported to exhibit a high expression of VEGF. VEGF promotes mitosis of vascular endothelial cells, dilates blood vessels, increases vascular permeability and induces the expression of a number of genes involved in the degradation of the vascular basement membrane (27–29). Tumors that exhibited a positive VEGF expression presented a worse prognosis (26).

Primary tumors cells recruit and induce the MSC differentiation residing locally in their origin sites. In addition, these tumors may release signals to induce the mesenchymal progenitor cells that circulate to extravasate and take up residence in the tumor stroma, and these tumor cells may also be induced to differentiate into various mesenchymal lineages. Previous findings indicate that a third of tumors release endocrine signals to impinge on the bone marrow, where these signals induce various types of stromal precursor cells to form and mobilize into the circulation, even prior to the mobilization of tumor cells into the circulation (30,31). Various types of tumors have an organ-specific preference for metastasis; while the metastatic behavior of OS varies, >80% of all OS metastasis arise in the lungs and other organs usually remain unaffected (32). The results of the present study showed that MSCs promote the pulmonary metastasis of OS, and the two cell types (MSCs and UMR-106) could interact with each other and increase the level of VEGF. These partly explain the mechanisms of metastasis of OS. However, why the metastasis has arisen in the lungs and how to modulate the expression of VEGF is unclear.

The present study demonstrated that MSCs promoted pulmonary metastasis following dissemination of UMR-106 and the level of VEGF increased in the UMR-106 and MSCs co-culture system. However, the steps of metastasis, whereby MSCs aid UMR-106 cells to achieve immune evasion within the circulatory system and how they interact with each other to upregulate the expression of VEGF, requires further investigation. These will help to develop strategies to block the OS invasion-metastasis cascade and to know the process occurring during the tumor cell dissemination from the primary site.

## Figures and Tables

**Table I:** Tumor metastatic rate.

Group	Time to autopsy^a^, weeks	Rats with macroscopic tumors, n	Rats with microscopic tumors, n
Control	5	0/10	0/10
UMR-106 (IT)	5	5/10	5/10
MSCs (IV)	5	0/10	0/10
UMR-106 (IT) and MSCs (IV)	5	6/10	6/10
UMR-106 (IV)	5	10/10	10/10
UMR-106 (IV) and MSCs (IV)	5	10/10	10/10

a Subsequent to tumor injection.

IT, injected through tibia; IV, injected through caudal vein; MSCs, mesenchymal stem cells.

## Abbreviations

CM-Dil: chloromethyl-dialkylcarbocyanine
MSCs: mesenchymal stem cells
OS: osteosarcoma
VEGF: vascular endothelial growth factor
